# Politics of citizenship during the COVID-19 pandemic: what can educators do?

**DOI:** 10.1186/s41018-020-00089-x

**Published:** 2021-01-06

**Authors:** Adeela Arshad-Ayaz, M. Ayaz Naseem

**Affiliations:** Concordia University, 1610 Ste Catherine Street West, Montreal, Quebec H3G1M8 Canada

**Keywords:** COVID-19, Citizenship, Education

## Abstract

As a once in a 100 years emergency, the COVID-19 pandemic has resulted in repercussions for the economy, the polity, and the social. Also, the ongoing pandemic is as much a teaching moment as it to reflect on the lack of critical citizenship education. The fault lines of the health system have become visible in terms of infection and death rates; the fault lines of the educational system are now apparent in the behavior of the citizens who are flouting the public health guidelines and, in certain cases, actively opposing these guidelines. The main objective of this commentary is to initiate a dialogue on the social contract between the state and the subjects and to see how education and educators can respond to the challenge of the new normal. It is contended that education under the new normal cannot afford to keep educating for unbridled productivity education under the new normal. It must have welfare, human connections, ethical relationships, environmental stewardship, and social justice front and center.

The global tally of confirmed COVID-19 infections has surpassed 35 million. The number of confirmed infections in Canada has reached the grim milestone of more than 175,000 infected, and more than 9000 deaths. COVID-19 is indeed once in a 100 years emergency. The pandemic has resulted in repercussions for the economy, the polity, and the social. The economy at all levels, from the local to the global, has taken a significant hit resulting in mass unemployment, colossal loss to the local and national economies, and disruption in supply chains all across the globe. In this commentary, we take the situation and the response to COVID-19 in Canada as a starting point to initiate a dialogue on the social contract between the state and the subjects and to see how education and educators can respond to the challenge of the new normal. It should also be noted that we use the notion of the ‘state’ to denote the neo-liberal state that took over from the welfare state in Canada (as it has globally). However, while our arguments and examples are mainly taken from Canada and to some extent from the US, the tendencies that we point out are present with varying degrees in a large number of countries.

At the political level, responses to the COVID-19 pandemic are varied, ranging from early, prompt, and decisive action (New Zealand, Australia, Singapore, Hong Kong, Israel) to initial denial that led to burgeoning infection and death rates (Great Britain, USA, Brazil, Russia). The former have more or less contained the pandemic, while the latter have massive losses in human and financial terms. This can be seen as the difference between leaders and managers. While the leaders took early decisive action and conveyed it to their publics through clear messaging, the managers delivered ambiguous (often contradictory) messages, politicized the response, and often ended up dividing the public rather than uniting it behind the cause of controlling the pandemic.

The success of any pandemic response depends, above all, on a complementary partnership between the state and the citizenry. Each of the partners is responsible for upholding their end of the contract. The continuing pandemic has, however, rendered visible the fault lines of the state-citizenship contract. With the exception of a handful of successful responses (stated above), states almost all over the world have largely followed the neo-liberal logic in handling the pandemic. For example, early evidence of the severity of the pandemic was ignored in order to keep the economies going. Messaging during this period bordered on bravado, and the actions were largely symbolic. Precious time to prepare was lost. Once the pandemic crossed the oceans and arrived on the North American shores, the states such as Canada and the USA responded by shifting the focus from the public good of pandemic management (state’s responsibility) to the individual goal of *infection-avoidance* (Maunula 2013). The shift obfuscated the complementarity between the two. The states governed by the managers *enforced* (albeit loosely) the accountability for individual acts of the citizens rather than holding the leaders accountable for the unpreparedness of the political elite and the health system (Boshra 2020). There was, for instance, no accountability for why the *stores of Personal Protective Equipment (PPE) in Ontario, Canada, had expired*? (Martell and Warburton 2020) Rather than admitting that the state has been negligent, the message from the state and health managers was that wearing of masks was *not only unnecessary but even hazardous for health* (Bruemmer 2020). The bottom line is that the state (at various levels) had neglected to prepare for a health emergency despite having *pandemic plans* and misled the public, and thus failed to slow down the spread of the pandemic (Health Canada n.d.). Messaging by the political and health authorities also did not acknowledge the tentativeness of the scientific and medical knowledge of the contagion.

It was, thus, up to the ‘pandemic citizen’ to slow down the pandemic. The pandemic subject was entrusted with finding information about the contagion (from a spate of confusing and contradictory messaging by the state along with a deluge of information on the internet) and ‘perform’ her/his ‘duty’ to ‘flatten the curve.’ Such articulation of the pandemic subject does not take into account the social complexities of society. There are, for example, those who cannot carry out obligations such as social distancing and personal hygiene due to their material conditions. On the other hand, there are those who do not do so because of their sense of entitlement. While both have been branded as COVIDIOTS (a pejorative term used for those who flout the public health directives), the difference between them is that of marginalization vs entitlement. The pandemic citizen is also now being called to help out with the system broken by years of neo-liberal neglect. Citizens are being cajoled into performing their ‘civic’ duty by *helping out with the neglected nursing and long-term care facilities* (Authier 2020a). The fragility of the health system and that of the nursing homes is not a product of the COVID-19 pandemic. It is but obvious that the public good of pandemic/health management is being transposed onto the pandemic citizen. The state is even ‘offering citizenship’ to immigrants willing to take on the burden of working as orderlies in the long-term care homes (Authier 2020b).

The COVID-19 citizen is both scared and confused. *The magnitude of the contagion* is what scares the pandemic citizen. The ambiguity of the messaging is what confuses her (Worldometers n.d.). Fright and confusion are compounded by social media. Couple this with the neo-liberal emphasis on individualism rather than the collective, and we have a situation where we see people behaving irrationally and endangering themselves and others. An overabundance of information (real, fake, conspiracy theories, science, and pseudo-science) on the internet and the inability of a vast majority of the populace to distinguish one from the other further complicates the situation. While the health system’s fault lines have become visible in terms of infection and death rates, the fault lines of the educational system are now apparent in the behavior of the citizens who are flouting the public health guidelines. Just as the pandemic has shown the effects of years of neglect and underfunding of the health care system, it has also made visible the effects of underfunding and neglect of the educational system, especially of disciplines and subjects related to teaching and learning about responsible citizenship.

In this sense, COVID-19 is as much a teaching moment as it to reflect on the lack of critical citizenship education. Under the pandemic, education systems have tried their best to do more with less. While the universities have hunkered down to save the semesters, the schools are now gearing up to reopen and save the school year (in many but not all jurisdictions). However, the main emphasis on education under the pandemic has been on delivery (remote, online, etc.). While the zeal, the spirit, and speed are impressive, educational institutions must also realize the need for a revamped vision of education (and citizenship) in the new ‘normal.’ COVID-19 is not only a medical emergency. It is as much a social, political, ethical, and social justice emergency. With the almost certainty of recurring waves of the COVID-19 pandemic, it is imperative that educators and educational institutions respond to the lesson learned from the first and second waves.

These lessons point to the need for equipping the populace with critical citizenship education with which it can raise crucial questions about the complementarity of the state-citizen contract. COVID-19 is a teachable moment. Education in the ‘new normal’ must move beyond the ‘boundless productivity’ paradigm and look towards educating a critical citizenry. Central to such an education is to inculcate the ability to question. In the context of the present pandemic, uncritical acceptance of the state narrative by the populace has emerged as the norm. Fewer questions have been raised on issues such as the dishonest messaging by the health authorities, incompetence of the public as well as the for-profit senior care facilities, disproportionate infection and death rates among the ethnic and racialized minorities, under or un-preparedness of the health and pharmaceutical infrastructures, incurring of massive national debt to bail out rich corporations, etc. Here, we must note the difference between criticality and reactionary opposition. It is, for example, argued by some that those who protest and demonstrate against the public health directives such as social distancing, wearing masks, etc. are also exercising their criticality by disputing governmental messaging. In our opinion, the resistance to public health directives is more oppositional and reactionary than critical. The opposition is based on political positions and affiliations rather than ethical, social justice, and public safety considerations. Critical citizenship and questioning must be based on the principle of ‘do no harm.’ If the exercise of ‘freedom’ and ‘choice’—two main arguments by the anti-mask, anti-social distancing advocates endangers or has the potential of endangering others, then it cannot be termed as behaviors resulting from critical thinking.

Education under the ‘new normal’ must inculcate the ability to question why the social contract between the state and the citizenry has broken? Education under the new normal must also pay attention to teaching about looking for connections and joining the dots. Learners have been stripped of this ability due to the compartmentalization of knowledge and subject areas. An essential question in this respect is, how can the stripped-down educational system take the role of inculcating critical citizenship values? Given the disinvestment from disciplines and subjects that are necessary to inculcate critical citizenship values among the students in favor of market-driven disciplines, the task for critical citizenship teachers is an uphill one. However, it does not need to remain this way in the future. As argued above, the pandemic has clearly shown the fault lines in educational systems that need to be taken into consideration immediately and over the long term. The dominant trend of disinvestment from subjects that impart skills necessary for critical citizenship has to be reversed. Similarly, the market-driven, productivity-focused education model must also be disrupted to usher in an ethics-based educational system with social justice at its heart.

Furthermore, critical citizenship education must instill reflexivity as a key educational value. Reflexivity is key to understanding our complicity in, or ignorance of, why the system is the way it is. In other words, reflexive thinking teaches one to question not only the system but also our place (and complicity) in the system and its consequences. In order to educate for reflexivity and reflexive citizenship, it is imperative to bring back (where they have been taken away) and/or introduce (where they are not present) subjects that may make the students think about their role (and/or complicity) in coloniality, race-relations, othering, environmental degradation and stewardship, gender relations, to name few. Finally, under the new normal, education has to focus on ethical action, ethical relationships, and social justice. Such refocusing has the prowess to stimulate engagement at the micro, mezzo, and macro levels of society both nationally and internationally. All of the above and more can be done by re-centering the human (as against economic) aims of education. The above-mentioned educational values cannot be taught by means of token, stand-alone courses on citizenship. They must be built into the educational ethos of educational institutions, policymaking, and society.

However, we are cognizant of the fact that such restructuring is a complex task. Years of neoliberal inroads into education cannot be corrected urgently. There still are questions that need to be asked, engaged upon, and answered before a restructuration can begin. These questions include but are not limited to (a) how can we reverse the trend of neo-liberal disinvestment in disciplines and subjects through which we can teach about critical responsible citizenship? (b) In the short term, given the constraints imposed by the neoliberal restructuring, how can we equip the educators to respond to the necessities of the ‘new normal’? Similarly, in the long term, how can we articulate an educational system that allows educators to work towards a critically educated citizenry well equipped to meet future challenges? (c) Educators and other stakeholders, collectively and individually, must start to think and engage about creating an educational system that is receptive to the teaching of subjects pertinent to reflexivity, critical consciousness, intersectionality, and relationality, to name a few.

Education under the new normal cannot afford to keep educating for unbridled productivity that has resulted in few profiting at the expense of many even during the pandemic. Education under the new normal must have welfare, human connections, ethical relationships, environmental stewardship, and social justice front and center.

## Data Availability

Not applicable.
